# Feasibility of virtual T2-weighted fat-saturated breast MRI images by convolutional neural networks

**DOI:** 10.1186/s41747-025-00580-3

**Published:** 2025-05-02

**Authors:** Andrzej Liebert, Dominique Hadler, Chris Ehring, Hannes Schreiter, Luise Brock, Lorenz A. Kapsner, Jessica Eberle, Ramona Erber, Julius Emons, Frederik B. Laun, Michael Uder, Evelyn Wenkel, Sabine Ohlmeyer, Sebastian Bickelhaupt

**Affiliations:** 1 Institute of Radiology, Universitätsklinikum Erlangen, Friedrich-Alexander-Universität Erlangen-Nürnberg (FAU), Erlangen, Germany; 2 Lehrstuhl für Medizinische Informatik, Friedrich-Alexander-Universität Erlangen-Nürnberg (FAU), Erlangen, Germany; 3 Institute of Pathology, University Regensburg, Regensburg, Germany; 4 Institute of Pathology, Universitätsklinikum Erlangen, Comprehensive Cancer Center Erlangen-EMN, Friedrich-Alexander-Universität Erlangen-Nürnberg (FAU), Erlangen, Germany; 5 Department of Gynecology and Obstetrics, Erlangen University Hospital, Comprehensive Cancer Center Erlangen-EMN, Friedrich-Alexander-Universität Erlangen-Nürnberg (FAU), Erlangen, Germany; 6 Medizinische Fakultät, Friedrich-Alexander-Universität Erlangen-Nürnberg (FAU), Radiologie München, München, Germany

**Keywords:** Artificial intelligence, Breast neoplasms, Diagnostic imaging, Magnetic resonance imaging, Neural network (computer)

## Abstract

**Background:**

Breast magnetic resonance imaging (MRI) protocols often include T2-weighted fat-saturated (T2w-FS) sequences, which support tissue characterization but significantly increase scan time. This study aims to evaluate whether a 2D-U-Net neural network can generate virtual T2w-FS (VirtuT2w) images from routine multiparametric breast MRI images.

**Methods:**

This IRB-approved, retrospective study included 914 breast MRI examinations from January 2017 to June 2020. The dataset was divided into training (*n* = 665), validation (*n* = 74), and test sets (*n* = 175). The U-Net was trained using different input protocols consisting of T1-weighted, diffusion-weighted, and dynamic contrast-enhanced sequences to generate VirtuT2. Quantitative metrics were used to evaluate the different input protocols. A qualitative assessment by two radiologists was used to evaluate the VirtuT2w images of the best input protocol.

**Results:**

VirtuT2w images demonstrated the best quantitative metrics compared to original T2w-FS images for an input protocol using all of the available data. A high level of high-frequency error norm (0.87) indicated a strong blurring presence in the VirtuT2 images, which was also confirmed by qualitative reading. Radiologists correctly identified VirtuT2 images with at least 96% accuracy. Significant difference in diagnostic image quality was noted for both readers (*p* ≤ 0.015). Moderate inter-reader agreement was observed for edema detection on both T2w-FS images (κ = 0.49) and VirtuT2 images (κ = 0.44).

**Conclusion:**

The 2D-U-Net generated virtual T2w-FS images similar to real T2w-FS images, though blurring remains a limitation. Investigation of other architectures and using larger datasets is necessary to improve potential future clinical applicability.

**Relevance statement:**

Generating VirtuT2 images could potentially decrease the examination time of multiparametric breast MRI, but its quality needs to improve before introduction into a clinical setting.

**Key Points:**

- Breast MRI T2w-fat-saturated (FS) images can be virtually generated using convolutional neural networks.
- Image blurring in virtual T2w-FS images currently limits their clinical applicability.
- Best quantitative performance could be achieved when using full dynamic-contrast-enhanced acquisition and DWI as input of the neural network.

**Graphical Abstract:**

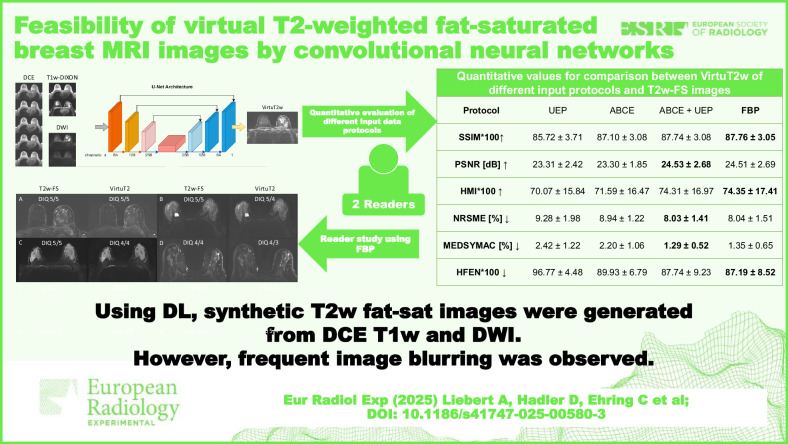

## Background

Breast magnetic resonance imaging (MRI) commonly utilizes a multiparametric protocol [1], which typically comprises different imaging sequences: unenhanced T1-weighted (T1w), T2-weighted fat-saturated (T2w-FS), diffusion-weighted imaging (DWI, with at least two *b*-values) and multiple T1w contrast-enhanced (CE) sequences that form the dynamic contrast-enhanced series.

Among those acquisition sequences, the T2w-FS acquisition provides morphological insight into tissue composition and fluid presence, aiding in lesion characterization and being included in classification schemes such as the Kaiser score [1]. However, acquiring the T2w-FS images with sufficient spatial resolution can significantly contribute to the total scanning time, sometimes accounting for up to 20% of the entire breast MRI examination [2, 3]. This influences scanner throughput, which is of relevance, especially when aiming to use breast MRI as a screening modality in screening programs [4].

In recent years, several publications have shown that deep learning approaches are technically feasible to derive “virtual” contrast-enhanced images from multiparametric unenhanced breast MRI acquisitions [5–10]. The aim of these virtual contrast-enhanced techniques, in all body regions, is focused on the reduction or even potential elimination of gadolinium-based contrast agent administration [11] in order to reduce related costs, potential side effects [12], and environmental issues [13]. These methods show the potential to synthesize additional or missing [14–16] diagnostic information from existing MRI acquisitions, thus potentially streamlining the imaging process.

This feasibility study builds on these recent works by investigating if a 2D-U-Net architecture might be used to generate images mimicking T2w-FS images from other breast MRI sequences such as T1w, DWI, and dynamic contrast-enhanced series. We evaluate such virtual T2w-FS mimicking (VirtuT2w) images by quantitative analysis of image similarity and error metrics as well as a multi-reader qualitative assessment.

## Methods

### Patient cohort and acquisition protocol

This retrospective study has been approved by the Ethics Committee of Friedrich-Alexander University Erlangen-Nurnberg waiving the need for informed consent. All included breast MRI examinations were clinically indicated routine examinations, performed between January 2017 and June 2020 at the Institute of Radiology, University Hospital Erlangen. The clinical indications included preoperative exclusion of multifocal disease, screening in women with a positive family history of breast cancer, exclusion of recurrent breast cancer, and clarification of unclear findings in mammography, ultrasound or due to clinical complaints.

The examinations were performed using one of two routine 3-T scanners (Magnetom Skyra Fit or Magnetom VIDA, Siemens Healthineers, Erlangen, Germany). Routine multiparametric protocol included unenhanced T1w-Dixon, T2w-FS, and DWI (*b*-values of 50, 750, and 1,500 s/mm^2^) acquisitions together with five T1-weighted contrast-enhanced dynamic acquisitions performed at 60 s interval after the intravenous administration of gadolinium-based contrast agent (gadobutrol, Bayer, Leverkusen, Germany; dose: 0.1 mmol/kg body weight; injection speed: 2 mL/s). Due to artifacts caused by the silicone on DWI acquisitions, the presence of breast implants was defined as an exclusion criterion. Detailed MRI acquisition parameters are presented in Table 1.

**Table 1:** Breast MRI protocol

Sequence abbreviated name	Sequence type	Matrix size	Field of view (mm^2^)	Slice thickness (mm)	Repetition time (ms)	Echo time (ms)	Inversion recovery time (ms)	Flip angle (°)	Number of averages	Fat saturation
T1w*	3D-GRE	448 × 448 × 112–128	360 × 360–430 × 430	1.5–1.8	5.97	2.46	-	10	1	None
T1w-FS	3D-GRE	448 × 448 × 112–128	360 × 360–430 × 430	1.5–1.8	5.97	2.46	-	10	1	Dixon water-phase
T2w-FS	2D-SE	448 × 448 × 34–49	340 × 340–430 × 430	4	3,570–5,020	60, 70	230	60–108	1	STIR
DWI**	2D-IR-DWI-EPI	256 × 160–200 × 34–49	350 × 219–430 × 269	4	6,290–9,660	66, 70	220, 250		3/8/20 or 3/8/15***	STIR

*DWI* Diffusion-weighted imaging, *GRE* Gradient echo, *SE* Spin-echo, *EPI* Echo-planar imaging, *STIR* Short tau inversion recovery

* Acquired before and during the five timepoints after intravenous injection of gadobutrol (Bayer, Leverkusen, Germany) 0.1 mmol/kg/body weight, injection speed 2 mL/s

** Acquired using three *b*-values: 50, 750, and 1,500 s/mm^2^

*** Number of averages for DWI indicates the number of averages for each *b*-value. The first set of presented values is for the acquisition performed on Skyra-Fit and the second set for that performed on Magnetom VIDA

**Fig. 1:**
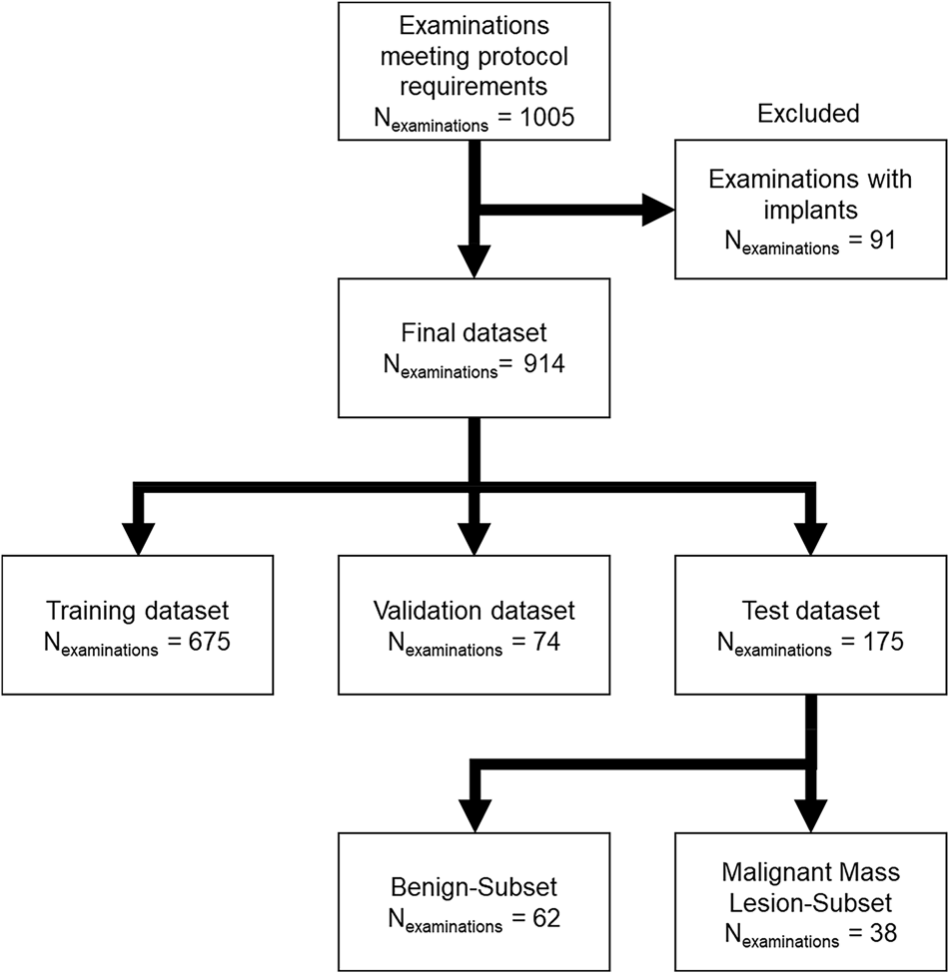
Study design flowchart. Protocol requirements for the examinations were defined as including uninterrupted acquisitions: T1-weighted, T1-weighted fat-saturated, T2-weighted fat-saturated, diffusion-weighted with multiple *b*-values including 50, 750, and 1,500 s/mm^2^ as well as a T1-weighted dynamic contrast-enhanced acquisition with five timepoints

Detailed definition of the final cohort based on the inclusion and exclusion criteria is presented in Fig. 1 (*n* = 816 patients, median age 50 interquartile range 43–59). The dataset was randomly divided at the examination level into a training (*n* = 665), validation (*n* = 74) and independent test set (*n* = 175). During the split, it was ensured that no data leakage at the patient level occurred between the datasets. Optimization of the training hyperparameters was performed based on the performance on the validation set. The independent test set was kept separately until the fixed model was deployed for the evaluation of this study.

### Data preprocessing

MRI acquisitions from each patient were extracted from the clinical Picture Archiving and Communication System and transferred to a local workstation for preprocessing using Python (version 3.9.10) with SITK framework (version 2.2.1) as follows. The field of view of the sequences was reduced to the smallest common volume among all of the sequences of the respective examination (in most cases, this was the field of view provided by the DWI). Furthermore, the 2D matrix, slice thickness and number of slices of all datasets were resampled to the acquisition of with the highest resolution, which was the T1w Dixon acquisition. Such registration ensures full correlation of voxel information among all acquisitions. Further, all acquisitions were z-score normalized, clamped at values of [-1, 15] and minimum-maximum rescaled, for the input images, to a domain [0, 1] and for the T2w-FS acquisition to a domain of [-1, 1]. Binary masks of the breast volume were generated using an in-house developed algorithm as presented in the Supplementary material.

### Neural network architecture and training

**Fig. 2:**
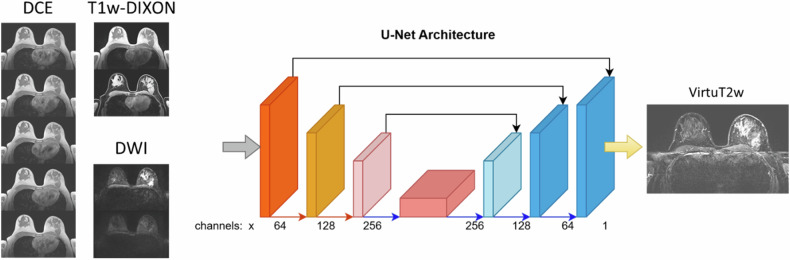
2D-U-Net architecture used for generating virtual T2-weighted fat-saturated (VirtuT2w) images. The network comprises three encoder stages (in blue) and three decoder stages (in orange) connected by a bottleneck stage (in red). The input of the network used during training consists of the T1-weighted acquisition (both with and without fat saturation), a series of T1-weighted contrast-enhanced acquisition, and of the diffusion-weighted acquisitions with *b*-values of 50 and 750 s/mm^2^. The inputs of the network are passed in the initial stage through a 1 × 1 convolution layer. The initial encoder stage generates *n* = 64 features; the feature number are multiplied by 2 after each encoder stage and the bottleneck resulting in a *n* = 512 maximal number of features. A skip connection between the respective levels of the encoder and decoder stages is introduced by concatenating the feature maps of the respective encoder stage to the input of the decoder stage. Between the encoder stages, the downsampling of the image dimensions is performed using a 2 × 2 convolution with stride of 2. The upsampling between the decoder stages is performed using a transposed 2 × 2 convolution. The 2nd and 3rd encoder stage as well as the bottleneck and the decoder stage consist of two sets of 3 × 3 convolution layer followed by batch normalization, dropout (with probability of 0.5), and leaky-rectified-linear-activation layers. The final encoder stage is followed by a 1 × 1 convolution layer and a tanh (hyperbolic tangent) activation function

A 2D-U-Net network was implemented using Python with PyTorch, MONAI and Lightning frameworks, as depicted in Fig. 2, similar to [10].

During training, single slices of the T1w Dixon in-phase image, T1w Dixon water-fraction image, DWI acquisitions with *b*-values of 50 and 750 s/mm^2^, and all five T1w dynamic contrast-enhanced acquisitions were used as input channels of the neural network. The corresponding slices of the T2w-FS acquisition were selected as the ground truth. Training was performed using as input four different clinically relevant breast MRI protocols in order to investigate the impact of these protocols on the ability to generate the VirtuT2w images. The four evaluated protocols were:

1. Unenhanced protocol (UEP), usually consisting of a T2w and DWI acquisitions [17] (only the DWI acquisitions with *b*-value of 50 and 750 s/mm^2^ were used as input);
2. Abbreviated contrast-enhanced protocol (ABCE), consisting of just the initial T1w Dixon acquisition and the 2nd time point of the dynamic acquisition [18];
3. ABCE + UEP [19];
4. Full breast protocol (FBP), consisting of the initial T1w-Dixon acquisition, full dynamic contrast-enhanced series as well as the DWI acquisition [1].

All trainings were performed using a loss function which combines L1-norm with structural similarity measure [20]:

$$L\left(\theta \right)=\left(1-{SSIM}(y,{y}_{i}^{\prime} )\right)+L1({y}_{i},{y}_{i}^{\prime} )$$

Training was performed on a dedicated workstation using a single NIVIDIA V100 Graphics Processing Unit (32GB RAM). For optimization, an ADAM optimizer was used with a learning rate of 10^-3^. Training was performed for *n* = 35 epochs using an *n* = 32 batch size.

### Test set evaluation

After the training, virtual T2w-FS images were generated using the independent test dataset. The resulting images were then evaluated quantitatively and in a reader study, performed by two board-certified radiologists.

### Quantitative evaluation

Quantitative metrics evaluated within the full breast volume for the VirtuT2w images were as follows: structural similarity index [21], peak signal-to-noise ratio, histogram mutual information, normalized root mean square error, median symmetric accuracy, and high-frequency error norm. All of the metrics were evaluated over the volume of the whole breast including breast muscle. Duration of the image generation by the network was evaluated using “tqdm” library (version 4.66.5) Python framework on NVIDIA DGX station (Linux Ubuntu 20.2, Intel Xeon 2.20 GHz 48 Cores, 256GB RAM) using a single V100 Graphics Processing Unit card.

### Qualitative multi-reader study

To supplement the quantitative analysis, a multi-reader study was performed on two subsets of the independent test set. This subset analysis was performed only for the best-performing input acquisition protocol as evaluated in the quantitative evaluation process.

The two subsets were randomly selected from the full test set as follows:

- Subset A (*n* = 38 examinations) comprising breast MRI examinations with histopathologically confirmed malignant mass lesions;
- Subset B (*n* = 62 examinations) comprising breast MRI examinations with benign findings as identified either by histopathology or based on the radiological assessment (if they were unequivocally benign in known to be benign by longitudinal assessments in follow-up examinations) only.

For both subsets, a reader study was performed by two board-certified radiologists: Reader 1, D.H., with 15 years of experience in breast MRI; and Reader 2 (S.B.), with 10 years of experience in breast MRI. The readers performed two reading sessions for each subset with at least two weeks between each reading session.

During each reading session, each radiologist evaluated an FBP of all cases from the respective subset. Of these cases, half included as part of the protocol VirtuT2w images and the other half included the original T2w-FS images. The radiologists were not informed about the number of cases with VirtuT2w or T2w-FS they would see in each session. The order in which the cases were shown was also randomized but the readers were aware whether they were evaluating subset A or B. During the reading, the following tasks were performed:

1. Define whether the presented image overall provides T2w-FS image characteristics (*e.g*., saturated appearance of fat, hyperintense depiction of fibroglandular tissue proportions, hyperintense depiction of cysts), answering the question with Yes or No;
2. Answer whether the presented image was an original T2w-FS acquisition or a VirtuT2w image;
3. Assess the diagnostic image quality (DIQ) using a 5-point Likert-like scale using the following scores in similarity to Chung et al [6]: 5 = excellent (acceptable for diagnostic use), 4 = good (acceptable for diagnostic use), 3 = acceptable (acceptable for diagnostic use but with minor issues), 2 = poor (not acceptable for diagnostic use), or 1 = very poor (not acceptable for diagnostic use). For cases that were rated to have an unacceptable diagnostic quality (scores 1 and 2), the readers were asked to indicate the reason for such rating as: artifact presence, image resolution, image blurring, lack of signal intensity in target structures, or others.

Additionally, for the first subset that included malignant mass lesions, in similarity to Kaiser score evaluation [22], the readers were asked if perilesional edema around the largest lesion was present.

### Statistical analysis

Normality of the quantitative metrics was investigated using Shapiro–Wilk test. Statistical evaluation of the differences in quantitative metrics between the different input data protocols was performed using Friedman repeated measures analysis of variance on ranks. *Post hoc* pairwise testing was performed using the Tukey test.

Statistical differences in the ordinal evaluations between the T2w-FS and VirtuT2w images were investigated using Wilcoxon signed rank test for each of the readers and a *p*-value lower than 0.05 was considered significant. Cohen κ analysis was applied for evaluating inter-reader agreement for DIQ and inter and intra-reader for the presence of edema among T2w-FS and VirtuT2w images.

Statistical testing was performed using SigmaPlot (v. 15.0), while evaluation of the inter-rater agreement was performed using Python (version 3.9.13) Sci-Kit Learn framework (version 1.5.2)

## Results

### Patient cohort characteristics

Detailed information about the full patient cohort can be found in Table 2.

**Table 2:** Demographics of the training/validation and independent test cohorts

Dataset	Full	Training and validation	Training	Validation	Independent test
Number of patients	816	641	567	74	175
Number of examinations	914	739	665	74	175
Split ratio of examinations (%)	100	80.9	72.8	8.1	19.1
Age (years)	50 [16]	50 [15]	50 [15]	51 [14]	51 [17]
BI-RADS score					
0	20 (2.2%)	16 (2.1%)	14 (2.1%)	2 (2.7%)	4 (2.3%)
1	3 (0.3%)	2 (0.02%)	2 (0.3%)	0 (0.0%)	1 (0.6%)
2	440 (48.1%)	364 (49.3%)	328 (49.3%)	36 (48.7%)	76 (43.4%)
3	70 (7.7%)	56 (7.6%)	53 (8.0%)	3 (4.0%)	14 (8.0%)
4	106 (11.6%)	78 (10.6%)	68 (10.2%)	10 (2.2%)	28 (16.0%)
5	64 (7.0%)	53 (7.2%)	50 (7.5%)	3 (13.5%)	11 (6.3%)
6	211 (23.1%)	170 (23%)	150 (22.6%)	20 (27.0%)	41 (23.4%)

The BI-RADS score refers to the highest score given for an examination during routine clinical reading. Age is presented as median [interquartile range]. Percentages represent the percentage of examinations in the respective dataset

The independent test set consisted of 175 cases, 66 malignant (38 mass and 28 non-mass lesions) and 109 benign. All these data were used for quantitative performance evaluation of the neural network. Table 3 shows the frequency of Breast Imaging Reporting and Data System (BI-RADS) adapted fibroglandular tissue and background parenchymal enhancement classes in the independent test set.

**Table 3:** Breast tissue type and background parenchymal enhancement in the independent test set (*n* = 175)

Breast tissue type		Background parenchymal enhancement	
Almost entirely fat	23 (13%)	Minimal	62 (35%)
Scattered	34 (19%)	Mild	40 (22%)
Heterogeneously dense	40 (22%)	Moderate	32 (18%)
Extremely dense	78 (44%)	Marked	41 (23%)

Categories attributed given according to the BI-RADS reporting recommendations

From the independent test set, a subset of 100 cases was created to perform the reader study (Fig. 1). The reader study subset consisted of all 38 cases with malignant mass lesion and *n* = 62 benign cases picked at random. Among the 38 cases with mass lesion, 35 were identified as a breast cancer of no special type, two as ductal carcinoma *in situ* and one as a mucinous carcinoma. Median size of the malignant mass lesions was 15.8 mm with an interquartile range of 9.1–20.4 mm (*n* = 11 cases with lesion size < 10 mm, *n* = 11 cases with lesion size > 20 mm).

### Evaluation of the VirtuT2w images

#### Quantitative evaluation

Table 4 shows the median and interquartile range of the quantitative performance metrics for the VirtuT2w images in comparison to the original T2w-FS for the four input data protocols. The best performance in regard to the structural similarity index, peak signal-to-noise ratio and high-frequency error norm was observed for the FBP. In regard to histogram mutual information, normalized root mean square error and median symmetrical accuracy, the best performance was observed for the ABCE + UEP protocol. Between the FBP and ABCE + UEP protocols and the UEP and ABCE protocols, significant differences could be observed for all of the quantitative metrics (all *p* < 0.001).

**Table 4:** Quantitative values for comparison between VirtuT2w images from different input protocols and T2w-FS images

Metrics/protocol	UEP	ABCE	ABCE + UEP	FBP
SSIM*100↑	85.72 [3.71]	87.10 [3.08]	87.74 [3.08]	**87.76 [3.05]**
PSNR [dB] ↑	23.31 [2.42]	23.30 [1.85]	**24.53 [2.68]**	24.51 [2.69]
HMI*100 ↑	70.07 [15.84]	71.59 [16.47]	74.31 [16.97]	**74.35 [17.41]**
NRSME [%] ↓	9.28 [1.98]	8.94 [1.22]	**8.03 [1.41]**	8.04 [1.51]
MEDSYMAC [%] ↓	2.42 [1.22]	2.20 [1.06]	**1.29 [0.52]**	1.35 [0.65]
HFEN*100 ↓	96.77 [4.48]	89.93 [6.79]	87.74 [9.23]	**87.19 [8.52]**

Bold metrics indicate best performance across the respective metric. All metric values are provided as median [interquartile range] as calculated on the whole independent test set. ↓ indicates that lower values suggest improvement of network performance; ↑ indicates that higher values suggest improvement of network performance

*ABCE* Abbreviated contrast-enhanced protocol, *ABCE* + *UEP* Abbreviated contrast-enhanced protocol plus diffusion-weighted acquisitions, *FBP* Full breast protocol, *HFEN* High-frequency error norm, *MEDSYMAC* Median symmetrical accuracy, *NRMSE* Normalized root mean square error, *PSNR* Peak signal-to-noise ratio, *SSIM* Structural similarity index, *UEP* Unenhanced protocol (diffusion-weighted imaging with *b*-value of 50 and 750 s/mm^2^)

The differences were not significant between the ABCE + UEP and FBP protocols in regard to the structural similarity index (*p* = 0.992), peak signal-to-noise ratio (*p* = 0.998), histogram mutual information (*p* = 0.987), normalized root mean square error (*p* = 0.394) and median symmetrical accuracy (*p* = 0.370). However, for the high-frequency error norm, the difference between the ABCE + UEP and FBP protocols was significant (*p* < 0.001). As such, as the best-performing input protocol, the FBP was chosen for the purpose of the qualitative reading. Supplementary Figs. 2–4 show the input data, the original T2w-FS acquisition, and VirtuT2w images generated using the four different input data protocols.

The median computational time for the generation of the VirtuT2w images was 31 s [interquartile range: 2 s].

#### Identification of original and VirtuT2w images

**Fig. 3:**
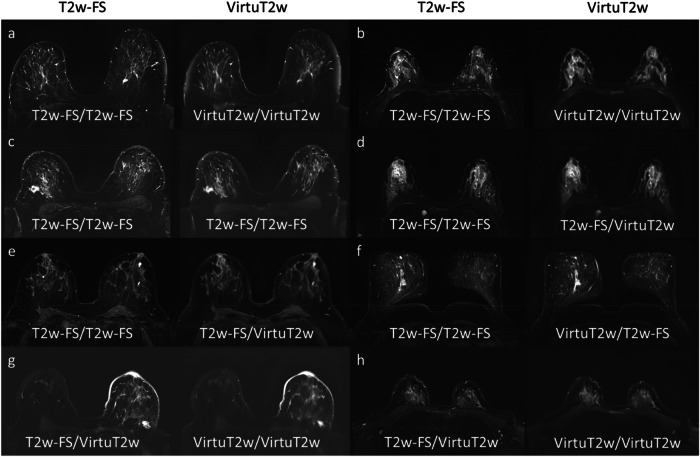
Example of image cases for which the two readers answered whether the respective image series looks like an original T2-weighted fat-saturated (T2w-FS) acquisition or a synthetic T2w-FS (VirtuT2w) image. The image names below each image indicate the respective readers’ answers, with Reader 1 presented on the left side and Reader 2 on the right side. In one case (**c**), both the readers mistook a VirtuT2w image for a T2w-FS. Notably, this image has a low level of blurring. In two cases (**d**, **e**), Reader 1 mistook the VirtuT2w image for a T2w-FS. In two cases, Reader 2 (**g**, **h**) mistook an original T2w-FS image for a VirtuT2w image. Both readers were able to identify the VirtuT2w images in most cases where significant blurring in the image was observed (**a**, **b**, **f**)

All VirtuT2w images (100/100) and T2w-FS images (100/100) were unanimously classified by both readers as providing typical characteristics of T2w-FS acquisitions. Yet, both readers were able to reliably identify the VirtuT2w images, with both readers correctly identifying 95.0% (95/100) of them. Accuracy for classifying between original and VirtuT2w images was 97.0% (194/200) for R1 and 96.5% (193/200) for R2. Comparison of the T2w-FS and VirtuT2w images for both correctly and falsely identified cases by both readers are presented in Fig. 3.

#### Image quality and diagnostic assessment

Inter-reader agreement for the evaluation of DIQ showed fair agreement between the readers for both T2w-FS images (κ = 0.25) and VirtuT2w images (κ = 0.24) for the individual quality scores.

**Fig. 4:**
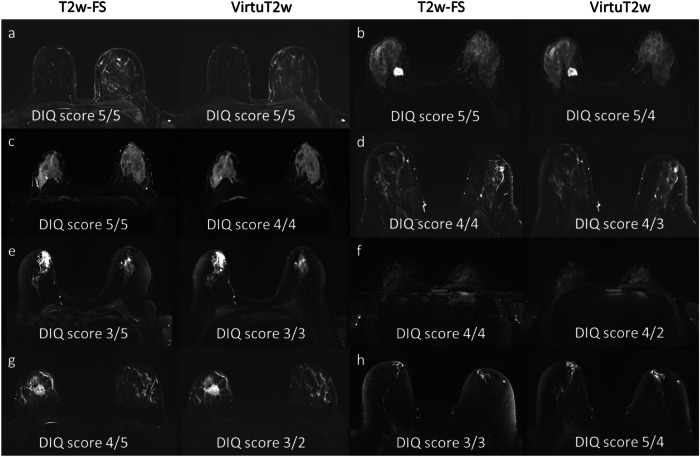
Example of image cases with DIQ ratings given by the two readers. The scores above each image indicate the respective reader’s answers, with Reader 1 presented on the left side and Reader 2 on the right side. In most cases (**a**–**e**), the DIQ was higher for the original T2-weighted fat-saturated (T2w-FS) images than for the virtual T2w-FS (VirtuT2w) images. An exception is in **h**, in which the fat saturation did not work as intended, and the DIQ of the T2w-FS was rated by both readers as only acceptable. In addition, two VirtuT2w images (**f**, **g**) were rated by Reader 2 as having a poor DIQ (score 2). No images in the whole cohort were rated as having a very poor quality. DIQ, Diagnostic image quality

**Fig. 5:**
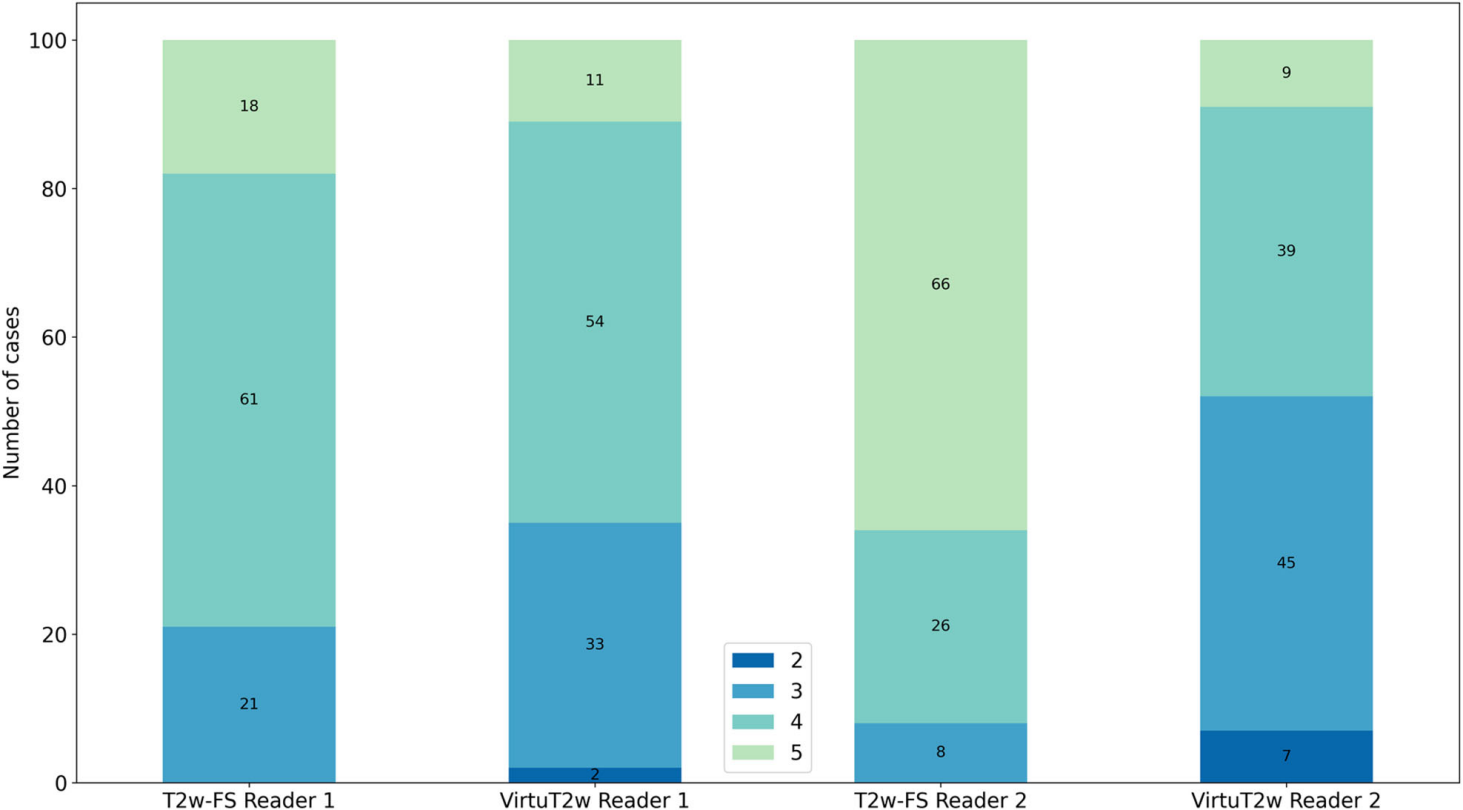
Frequencies of different DIQ scores given to the original T2-weighted fat-saturated (T2w-FS) and to the synthetic virtual T2w-FS (VirtuT2w) images by the two readers. For both readers, significant difference in the median of the score could be observed between the two methods (Reader 1, *p* = 0.015; Reader 2, *p* < 0.001). DIQ, Diagnostic image quality

Significant difference in the DIQ scores given were found for both readers between the T2w-FS and VirtuT2w images (Reader 1, *p* = 0.015; Reader 2, *p* ≤ 0.001) with VirtuT2w images showing a lower median for Reader 2 (T2w 5 *versus* VirtuT2w 3). Example of T2w-FS and VirtuT2w images with different DIQ scores are presented in Fig. 4. Frequencies of the DIQ scores for both T2w-FS acquisition and for VirtuT2w are presented in Fig. 5. For all evaluations with DIQ scores below 3 (Reader 1, 2 examinations; and Reader 2, 7 examinations), the reader indicated image blurring as reason for the score.

#### Identification of edema

With regard to the reading task of identification of the presence of edema, using the T2w-FS acquisitions, Reader 1 identified it in *n* = 19 cases and Reader 2 in *n* = 24 and both readers identified edema in *n* = 17 cases. At the same on the VirtuT2w images time, R1 identified the edema in *n* = 17 cases and R2 in *n* = 22, with both of the readers identifying it in *n* = 11 cases. Moderate inter-rater agreement could be observed for both T2w-FS acquisitions (κ = 0.49) as well as the VirtuT2w images (κ = 0.44) could be observed for this task. Between the T2w-FS and VirtuT2w images, fair intra-reader agreements could be observed for both Reader 1 (κ = 0.19) and Reader 2 (κ = 0.39). If the presence of edema was identified on the original T2w-FS acquisition, it could also be identified on the VirtuT2w images in only 53% (10/19) and 58% (14/24) of cases by Reader 1 and Reader 2, respectively.

**Fig. 6:**
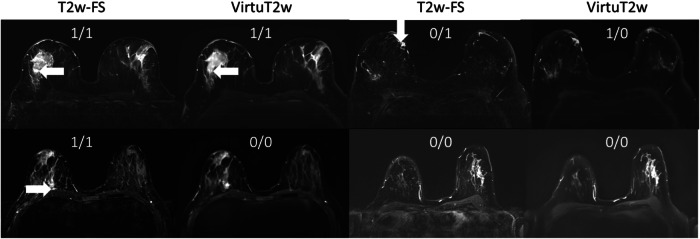
Example images of malignant cases with indications whether presence of edema was identified by Reader 1/Reader 2 respectively. A score of 1 indicates that edema was present. White arrows indicate regions where perilesional edema was identified

Figure 6 shows exemplary cases in which the presence of edema was identified on either the original T2w-FS acquisitions or on the VirtuT2w image.

## Discussion

This study demonstrates the technical feasibility of generating T2w-FS mimicking images from a multiparametric breast MRI protocol e using a 2D-U-Net architecture. The value of this result is due to the role of T2w sequences as part of multiparametric breast MRI protocols, described to aid in the characterization of findings (contributing to breast MRI specificity of, *e.g*., in women undergoing high-risk screening [23]) and considering the acquisition time needed. To our knowledge, this study is among the first [24] of its kind to show that such application of deep learning is possible.

Our results indicated the VirtuT2w images to comprise typical T2w-FS image characteristics and high quantitative similarity metrics. However, the appearance of the VirtuT2w images differed enough from the original T2w-FS images for the readers to be reliably identified with a very high accuracy of about 96–97%. Further, the reading revealed increased presence of blurring comprising DIQ in the VirtuT2w images.

With a structural similarity index of 0.87 and a peak signal-to-noise ratio of 24.90 dB, the quantitative performance of the generated VirtuT2w images was found to be slightly lower than the results achieved by a recently published approach of Zhang et al [24]. However, it should be noted that these two studies were performed on different datasets so that the results comparability is limited. Compared to this study, our study expanded the interpretability of the quantitative metrics by the inclusion of an evaluation of the high-frequency error norm, which is widely used to assess the level of image blurring [25]. Our study also confirmed the observation of the study performed by Zhang et al in regard to the input data necessary for the generation of the VirtuT2w images. Both studies indicate that the differences between the FBP and ABCE + DWI are negligible for most of the similarity and error metrics. However, interestingly, an investigation of the high-frequency error norm metric showed that in regard to this metric, the FBP performs better. This indicates that the high-frequency error norm metric could be potentially beneficial to the evaluation of virtually generated images in breast MRI.

Recent publications on breast MRI protocols indicated that T2w-FS acquisitions might take up to approximately 20% of the whole on-table examination time [2, 3]. Recent efforts in shortening the acquisition time of the T2w-FS acquisition show a potential decrease of the acquisition time by 48–58% [26, 27]. Nevertheless, our median computational time for the VirtuT2w images of 31 s is still below half of the reported acquisition times of T2w-FS with the application of deep learning reconstruction [26]. Whilst only presenting a first feasibility of the VirtuT2w images, this development shows potential to further streamline scanner throughput times without compromising diagnostic assessments. Additionally, a recent study indicates that even in a high throughput scenario enough time between examinations should be available to perform the computation of the VirtuT2w images [2].

However, these generated VirtuT2w images are not without flaws, and the current feasibility status is still far away from clinical applicability, hindered by the significant blurring of its generated images. The blurring significantly affected the overall DIQ scores for both readers. The high value of the high-frequency error norm of approximately 0.87 when using the FBP is well in agreement with the observed high level of blurring occurring in the VirtuT2w images. The blurring might be caused by the resolution of DWI as relevant information source for the network. Another reason for the blurring might be the applied U-Net architecture, as encoder-decoder architectures are known for their blurry outputs if large variations in shape and scale are present in target regions [28, 29]. Such a situation naturally occurs in T2w-FS images where large regions of fibroglandular tissue occur among areas speckled with small-scale nodular or linear appearing tissue tracks. Interestingly, the observed blurring appears in a similar fashion to blurring observed in the virtual contrast-enhanced example images presented by Chung et al [6]. This may point towards the architecture’s responsibility for the blurring as both this work and the work by Chung et al are using encoder-decoder architectures. Nevertheless, for a definitive answer on this aspect further investigations of other architectures, for example through inclusion of attention mechanisms [7, 8] or a complete change of the architecture [8] will be necessary.

Furthermore, the VirtuT2w images represented visually visible edema only in 53–58% of cases in which the edema was observed in the original T2w-FS acquisitions. However, this evaluation is as well dependent on the reader, as it can be seen from the only moderate inter-reader agreement as well as from the varying number of cases in which the edema was detected in the original acquisitions by each of the two readers. Additionally, due to its nature, this evaluation does not have a reference value against which it could be compared.

A further study limitation is found in the dataset of examinations performed on just two scanner types from a single manufacturer, with both scanners using 3-T magnetic field strength and a harmonized acquisition protocol with high-quality DWI acquisitions. As we did not perform an external validation due to missing fully matching datasets in public repositories, we cannot draw conclusions on generalizability across different MRI settings. These limitations affect the generalizability of our method, and further investigations of this method on larger external datasets should be performed in future studies. Our study also did not investigate additional more advanced neural network architectures as suggested by Zhang et al [24] as the aim was to investigate simple network architectures which do not use generative adversarial loss during the training. Based on the results from Zhang et al investigation of such architectures might allow to overcome the increased levels of blurring of our VirtuT2w images.

In conclusion, our study suggests that a neural network is technically able to generate images that mimic the contrast of T2w-FS acquisitions in breast MRI, however, with increased blurring as compared to acquired T2w-FS data. Further research on this topic is necessary to overcome the current limitations of this initial technical setup.

## Supplementary information

**Additional file 1:** **Supplemental Fig. S1.** Example of binary masks generated using the in-house algorithm of every 10th slice for three examinations (**a**, **b**, **c**). It can be noted that the masks include both breast volume as well as parts of the heart, thorax and lung. **Supplemental Fig. S2.** Exemplary case of a 67-year-old patient undergoing diagnostic MRI with a malignant mass lesion in the right breast (white arrow). Histopathology confirmed breast carcinoma nonspecific type. Images of the Input sequences are presented in top two rows, except for the T1w subtraction image of the second time point which is presented for the viewer’s reference. Original T2w-FS image and the results of VirtuT2w generated images using one of the four clinically relevant protocols are presented in the bottom row. Two findings (marked with white and yellow arrow) can be noted in the right breast on the T1w subtraction image. First finding is a mass-enhancing lesion (white arrow) which shows high signal intensities both in the T1w-CE images, in the T2w image as well as in the DWI acquisitions. Second finding (yellow arrow) shows non-mass-enhancement with lower signal intensity levels. This NME region might be considered as potentially indicative of malignancy due to the typical diffuse appearance of the edema around it in the T2w-FS image. Similar diffuse appearance of the edema can be observed in the VirtuT2w-ABCE+UEP image and in the VirtuT2w-FBP image. **Supplemental Fig. S3.** Exemplary case of a 67-year-old patient undergoing diagnostic MRI with a malignant mass lesion in the left breast (white arrow). Histopathology confirmed breast carcinoma non-specific type. Images of the Input sequences are presented in top two rows, except for the T1w subtraction image of the second time point which is presented for the viewer’s reference. Original T2w-FS image and the results of VirtuT2w generated images using one of the four clinically relevant protocols. Comparable hyperintensity patterns can be observed across all four VirtuT2w results with an increased level of blurring visible in both UEP and ABCE acquisitions. **Supplemental Fig. S4.** Exemplary case of a 47-year-old patient undergoing MRI as part of high-risk screening without any malignant visible lesions. High amount of dense fibroglandular tissue can be observed for the patient. Images of the input sequences are presented in top two rows, except for the T1w subtraction image of the second time point which is presented for the viewer’s reference. Original T2w-FS image and the results of VirtuT2w generated images using one of the four clinically relevant protocols. Limited hyperintensity of the breast parenchyma can be observed in the VirtuT2w – ABCE images while signal intensity of the breast parenchyma comparable to the original T2w acquisition can be observed in the all three other VirtuT2w images. Stronger image blurring can be observed in the VirtuT2w – UEP image. **Supplemental Fig. S5.** Exemplary case of a 51-year-old patient u undergoing MRI as part of high-risk screening without any malignant visible lesions. High amount of dense fibroglandular tissue can be observed for the patient. Images of the input sequences are presented in top two rows. Original T2w-FS image and the results of VirtuT2w generated images using one of the four clinically relevant protocols are presented in the bottom row. VirtuT2w -UEP image shows significant level of blurring. VirtuT2w – ABCE image shows a decreased level of breast parenchyma signal intensity in comparison to the original T2w-FS image as well as against the other three VirtuT2w images.

## Data Availability

Original image data used in this work are not publicly available to preserve individuals’ privacy under the European General Data Protection Regulation. The institution handling this data is the Institute of Radiology, University Hospital Erlangen.

## Abbreviations

ABCE: Abbreviated contrast-enhanced
DIQ: Diagnostic image quality
DWI: Diffusion-weighted imaging
FBP: Full breast protocol
T1w: T1-weighted
T2w-FS: T2-weighted fat-saturated
UEP: Unenhanced protocol
VirtuT2w: Virtual T2w-FS

## Declarations

### Study subjects or cohorts overlap

Some study subjects or cohorts have been previously reported in 10.1007/s00330-023-10469-7; 10.1007/s00330-022-08626-5; 10.1038/s41598-023-37342-3; 10.1101/2024.05.03.24306067.
