# Supplementary material for: Feasibility of virtual T2-weighted fat-saturated breast MRI images by convolutional neural networks
Source: Eur Radiol Exp. 2025 May 2;9:47. doi: 10.1186/s41747-025-00580-3 (PMC12048370; doi:10.1186/s41747-025-00580-3)
Supplement: Supplementary file 1 — Additional file 1: Supplemental Fig. S1. Example of binary masks generated using the in-house algorithm of every 10th slice for three examinations (a, b, c). It can be noted that the masks include both breast volume as well as parts of the heart, thorax and lung. Supplemental Fig. S2. Exemplary case of a 67-year-old patient undergoing diagnostic MRI with a malignant mass lesion in the right breast (white arrow). Histopathology confirmed breast carcinoma nonspecific type. Images of the Input sequences are presented in top two rows, except for the T1w subtraction image of the second time point which is presented for the viewer’s reference. Original T2w-FS image and the results of VirtuT2w generated images using one of the four clinically relevant protocols are presented in the bottom row. Two findings (marked with white and yellow arrow) can be noted in the right breast on the T1w subtraction image. First finding is a mass-enhancing lesion (white arrow) which shows high signal intensities both in the T1w-CE images, in the T2w image as well as in the DWI acquisitions. Second finding (yellow arrow) shows non-mass-enhancement with lower signal intensity levels. This NME region might be considered as potentially indicative of malignancy due to the typical diffuse appearance of the edema around it in the T2w-FS image. Similar diffuse appearance of the edema can be observed in the VirtuT2w-ABCE+UEP image and in the VirtuT2w-FBP image. Supplemental Fig. S3. Exemplary case of a 67-year-old patient undergoing diagnostic MRI with a malignant mass lesion in the left breast (white arrow). Histopathology confirmed breast carcinoma non-specific type. Images of the Input sequences are presented in top two rows, except for the T1w subtraction image of the second time point which is presented for the viewer’s reference. Original T2w-FS image and the results of VirtuT2w generated images using one of the four clinically relevant protocols. Comparable hyperintensity patterns can be obser [file 41747_2025_580_MOESM1_ESM.pdf]

Feasibility of virtual T2-weighted fat-saturated breast MRI images by convolutional neural networks

ELECTRONIC SUPPLEMENTARY MATERIAL

Binary mask generation

Binary masking was performed on the original T1w acquisition. Binary masks were calculated on multiple maximum intensity projections (MIP) in z-direction (slice direction). For each slice of the T1w data, an individual MIP was calculated. Those MIPs were binarized using a mean thresholding algorithm. To ensure homogeneous masks including the whole breast tissue, binary dilation with a disk-shaped kernel of 5 pixels in diameter followed by a binary closing operation was performed. To account for different anatomical properties in different slice locations of the volumetric data, MIPs were created using a selection of adjacent slices. The range of adjacent slices depending on the position in the volume is represented in Equation (1) below, where n_{slices} define the range of slices considered for the MIP generation for a specific position. The masks were stored in NIfTI format with the spacing, direction and origin of the T1w data to enable a visualization with medical visualization applications.

$$n_{slices}(slice) = \begin{cases} [0, 6] & if\ slice \leq 3 \\ [slice - 3, slice + 3] & if\ 3 < slice < 9 \\ [slice - 3, slice + 3] & if\ \max slice - 6 < slice < \max slice - 3 \\ [\max slice - 6, \max slice] & if\ slice \geq \max slice - 3 \\ [slice - 8, slice + 8] & otherwise \end{cases} \quad (1)$$

Supplemental Fig. S1 displays the results of breast volume masking on corresponding T1-weighted slices for a representative case. The masks successfully include the whole breast tissue while the air around the patient is excluded as well as parts of the thorax, and most of the lung and heart tissue.

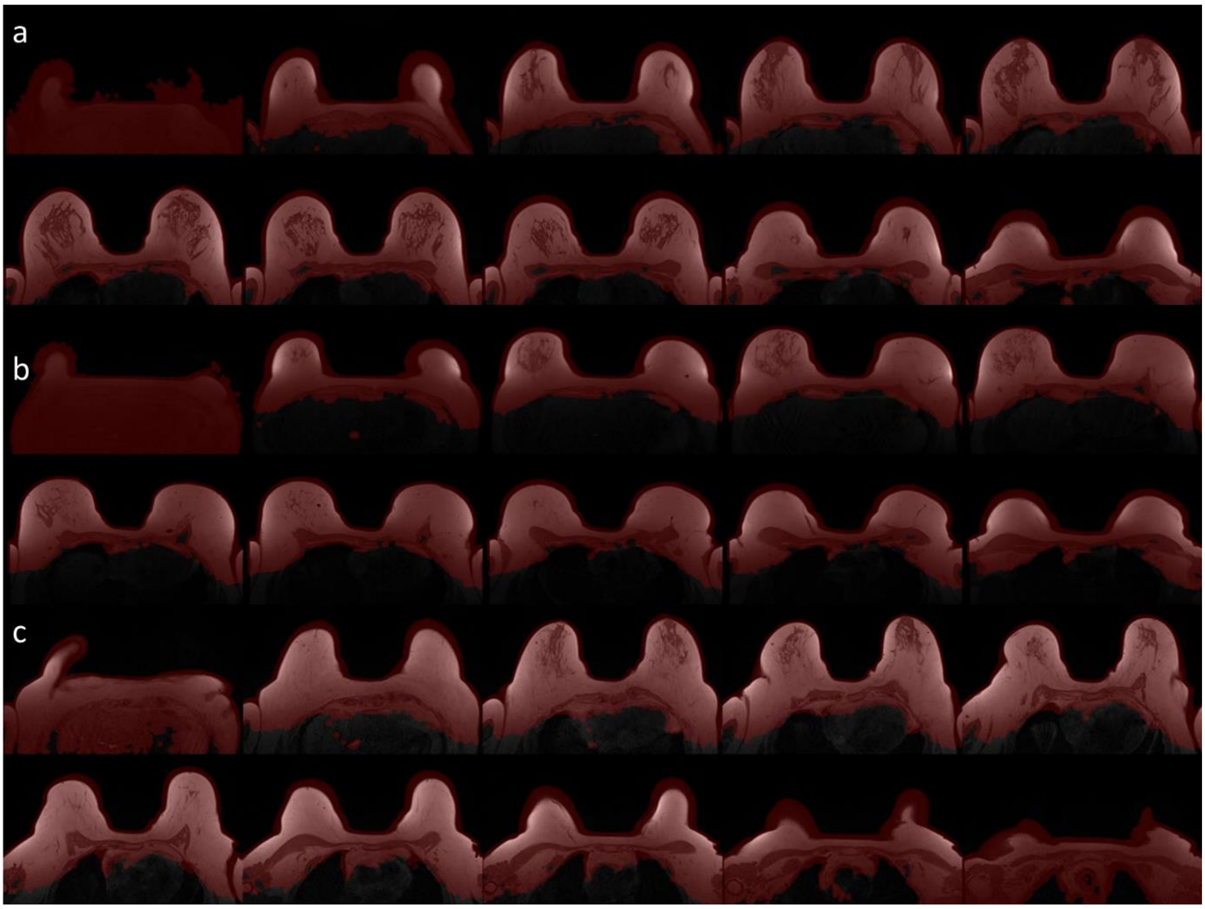

Supplemental Fig. S1. Example of binary masks generated using the in-house algorithm of every 10th slice for three examinations (**a**, **b**, **c**). It can be noted that the masks include both breast volume as well as parts of the heart, thorax and lung.

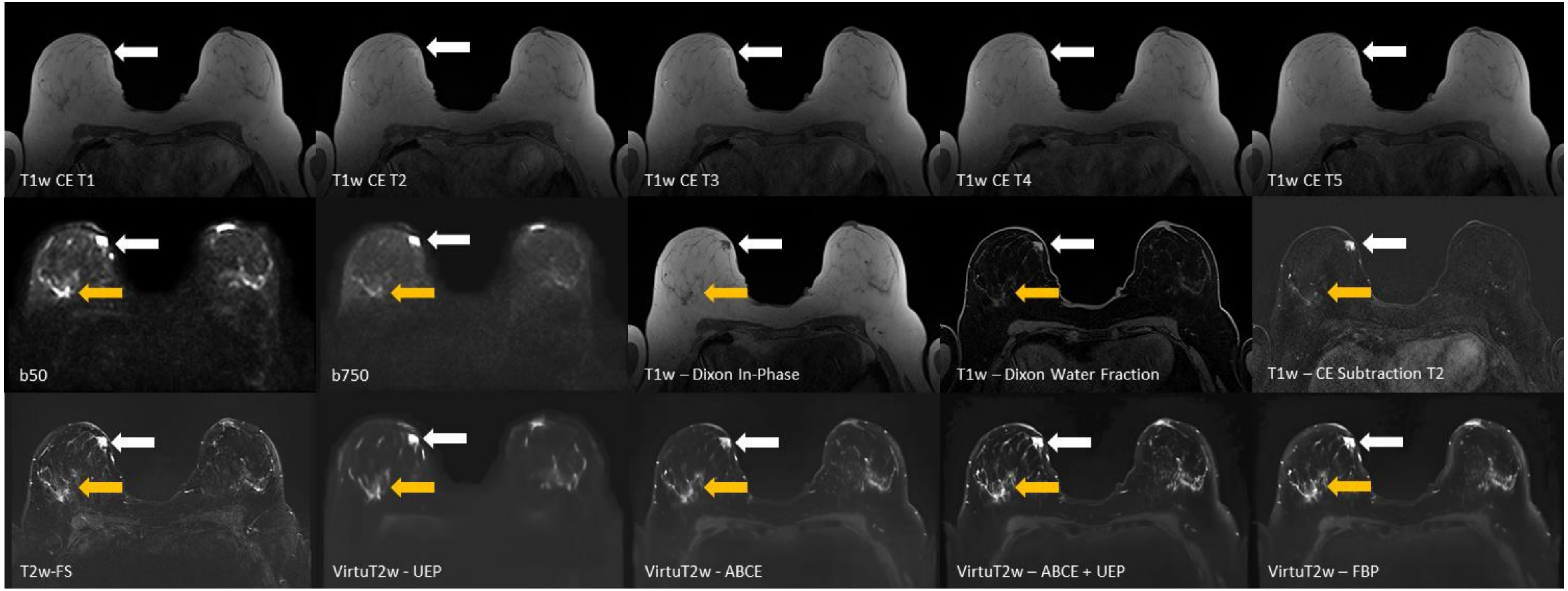

Supplemental Fig. S2: Exemplary case of a 67-year-old patient undergoing diagnostic MRI with a malignant mass lesion in the right breast (white arrow). Histopathology confirmed breast carcinoma nonspecific type. Images of the Input sequences are presented in top two rows, except for the T1w subtraction image of the second time point which is presented for the viewer's reference. Original T2w-FS image and the results of VirtuT2w generated images using one of the four clinically relevant protocols are presented in the bottom row. Two findings (marked with white and yellow arrow) can be noted in the right breast on the T1w subtraction image. First finding is a mass-enhancing lesion (white arrow) which shows high signal intensities both in the T1w-CE images, in the T2w image as well as in the DWI acquisitions. Second finding (yellow arrow) shows non-mass-enhancement with lower signal intensity levels. This NME region might be considered as potentially indicative of malignancy due to the typical diffuse appearance of the edema around it in the T2w-FS image. Similar diffuse appearance of the edema can be observed in the VirtuT2w-ABCE+UEP image and in the VirtuT2w-FBP image.

UEP- unenhanced protocol consisting of just DWI acquisition (b-values 50 and 750 s/mm^2), ABCE – abbreviated contrast enhanced protocol consisting of T1w-DIXON acquisition (both in-phase and Water Fraction) as well as of the contrast enhanced 2nd time-point acquisition (T1w CE T2), ABCE+UEP – ABCE protocol with addition of a DWI acquisition (b-values 50 and 750 s/mm^2), FBP – full breast protocol consisting of all input datasets.

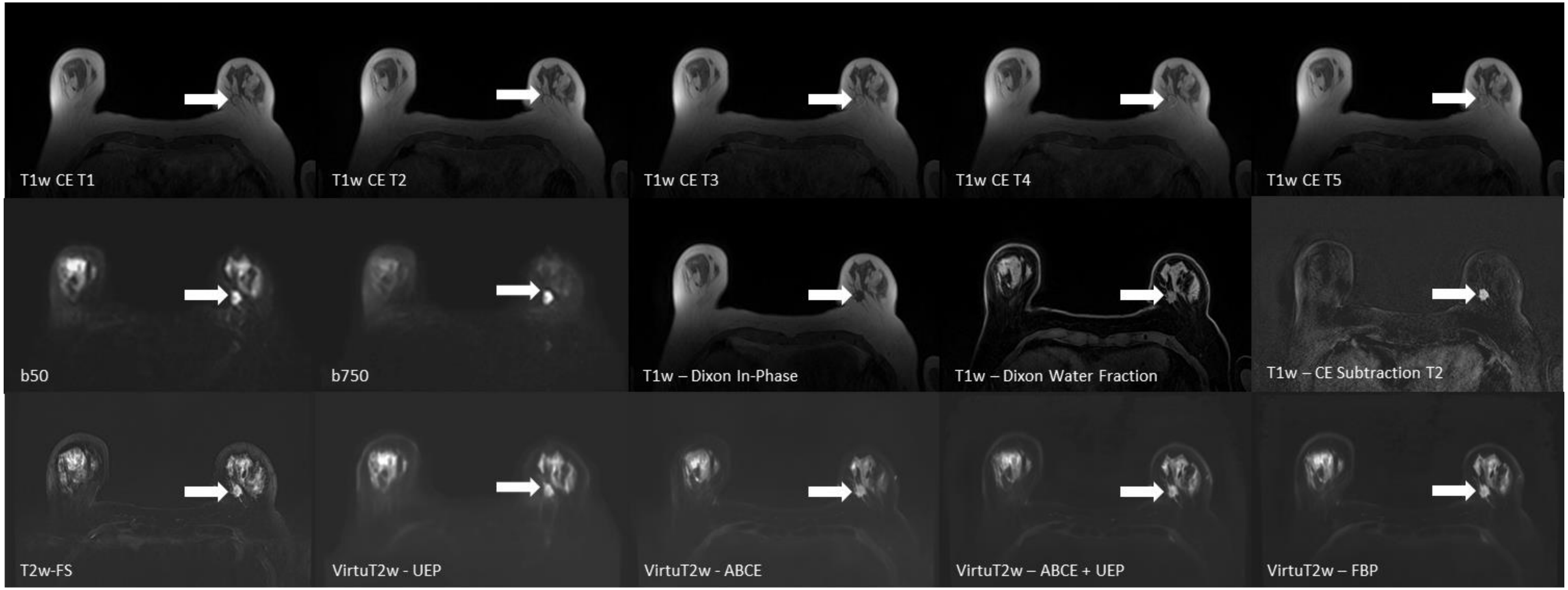

Supplemental Fig. S3: Exemplary case of a 67-year-old patient undergoing diagnostic MRI with a malignant mass lesion in the left breast (white arrow). Histopathology confirmed breast carcinoma non-specific type. Images of the Input sequences are presented in top two rows, except for the T1w subtraction image of the second time point which is presented for the viewer's reference. Original T2w-FS image and the results of VirtuT2w generated images using one of the four clinically relevant protocols. Comparable hyperintensity patterns can be observed across all four VirtuT2w results with an increased level of blurring visible in both UEP and ABCE acquisitions.

UEP- unenhanced protocol consisting of just DWI acquisition (b-values 50 and 750 s/mm^2), ABCE – abbreviated contrast enhanced protocol consisting of T1w-DIXON acquisition (both in-phase and Water Fraction) as well as of the contrast enhanced 2nd time-point acquisition (T1w CE T2), ABCE+UEP – ABCE protocol with addition of a DWI acquisition (b-values 50 and 750 s/mm^2), FBP – full breast protocol consisting of all input datasets.

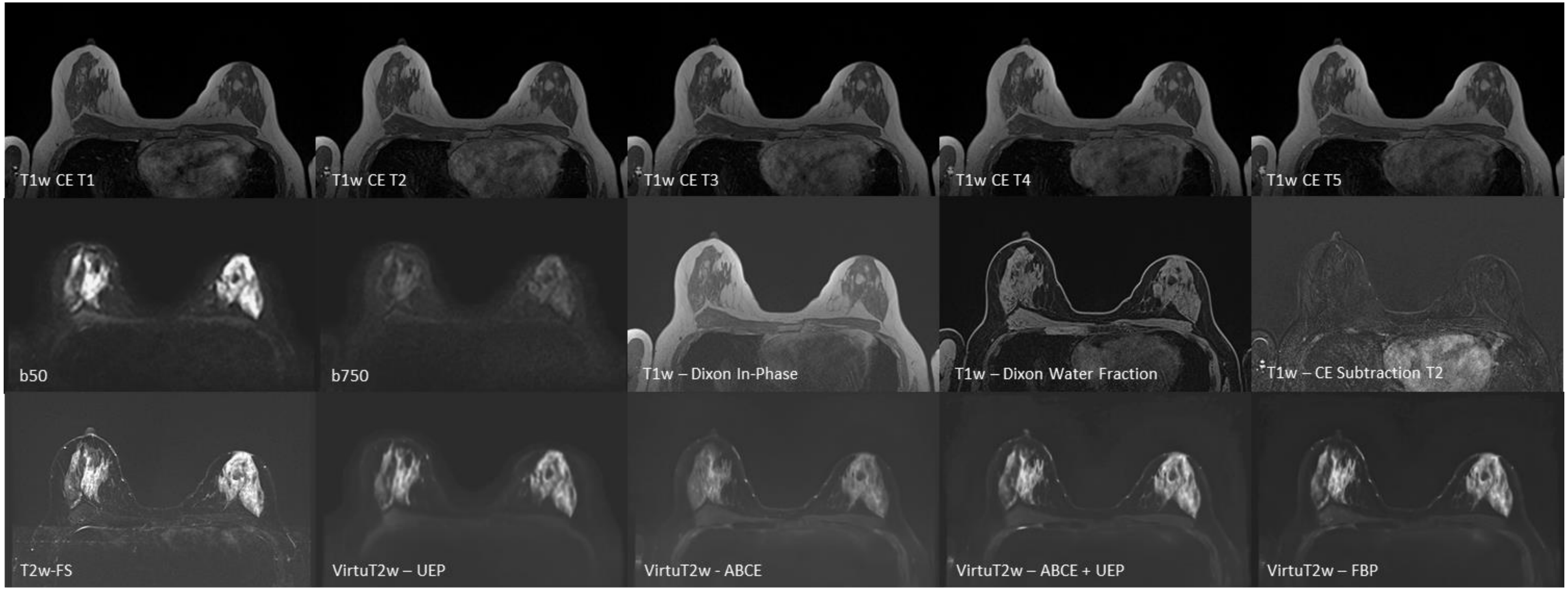

Supplemental Fig. S4: Exemplary case of a 47-year-old patient undergoing MRI as part of high-risk screening without any malignant visible lesions. High amount of dense fibroglandular tissue can be observed for the patient. Images of the input sequences are presented in top two rows, except for the T1w subtraction image of the second time point which is presented for the viewer's reference. Original T2w-FS image and the results of VirtuT2w generated images using one of the four clinically relevant protocols. Limited hyperintensity of the breast parenchyma can be observed in the VirtuT2w – ABCE images while signal intensity of the breast parenchyma comparable to the original T2w acquisition can be observed in the all three other VirtuT2w images. Stronger image blurring can be observed in the VirtuT2w – UEP image. UEP- unenhanced protocol consisting of just DWI acquisition (b-values 50 and 750 s/mm^2), ABCE – abbreviated contrast enhanced protocol consisting of T1w-DIXON acquisition (both in-phase and Water Fraction) as well as of the contrast enhanced 2nd time-point acquisition (T1w CE T2), ABCE+UEP – ABCE protocol with addition of a DWI acquisition (b-values 50 and 750 s/mm^2), FBP – full breast protocol consisting of all input datasets.

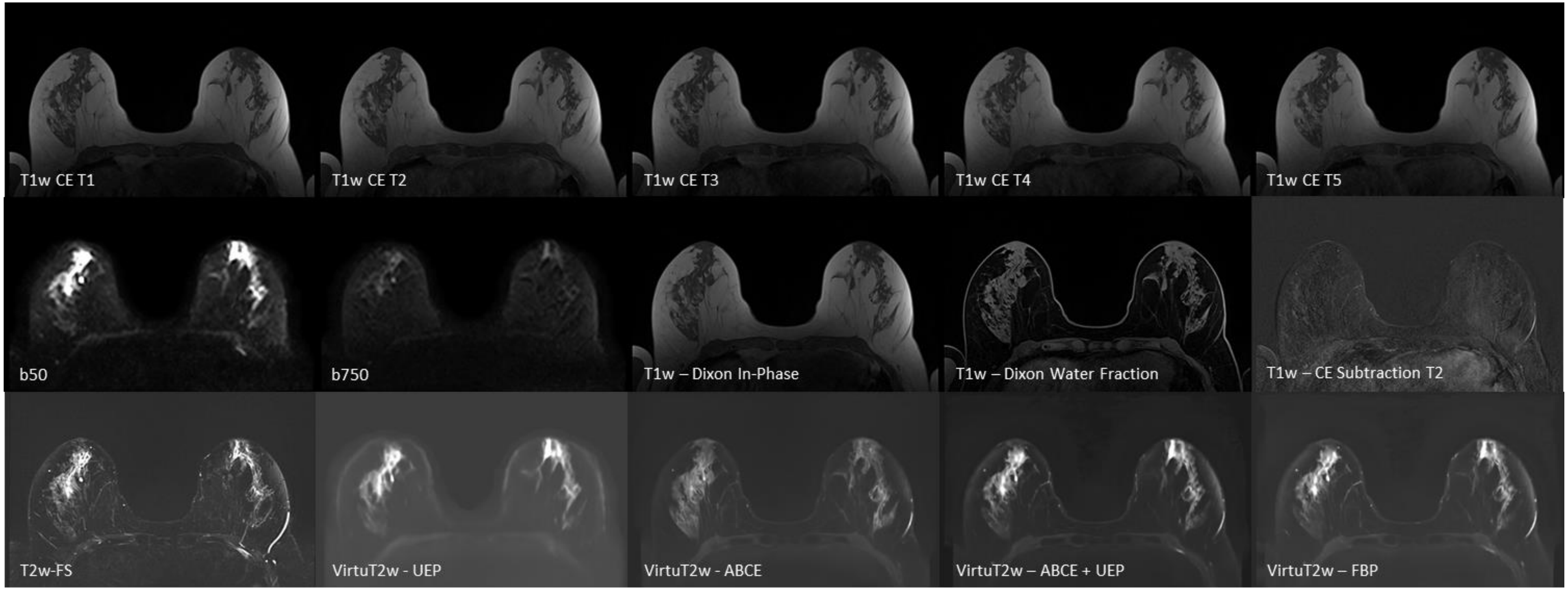

Supplemental Fig. S5: Exemplary case of a 51-year-old patient u undergoing MRI as part of high-risk screening without any malignant visible lesions. High amount of dense fibroglandular tissue can be observed for the patient. Images of the input sequences are presented in top two rows. Original T2w-FS image and the results of VirtuT2w generated images using one of the four clinically relevant protocols are presented in the bottom row. VirtuT2w -UEP image shows significant level of blurring. VirtuT2w – ABCE image shows a decreased level of breast parenchyma signal intensity in comparison to the original T2w-FS image as well as against the other three VirtuT2w images. UEP- unenhanced protocol consisting of just DWI acquisition (b-values 50 and 750 s/mm^2), ABCE – abbreviated contrast enhanced protocol consisting of T1w-DIXON acquisition (both in-phase and Water Fraction) as well as of the contrast enhanced 2nd time-point acquisition (T1w CE T2), ABCE+UEP – ABCE protocol with addition of a DWI acquisition (b-values 50 and 750 s/mm^2), FBP – full breast protocol consisting of all input datasets.
